# In-Vivo and Ex-Vivo Tissue Analysis through Hyperspectral Imaging Techniques: Revealing the Invisible Features of Cancer

**DOI:** 10.3390/cancers11060756

**Published:** 2019-05-30

**Authors:** Martin Halicek, Himar Fabelo, Samuel Ortega, Gustavo M. Callico, Baowei Fei

**Affiliations:** 1 Department of Bioengineering, The University of Texas at Dallas, 800 W. Campbell Road, Richardson, TX 75080, USA; martin.halicek@gatech.edu (M.H.); hfabelo@iuma.ulpgc.es (H.F.); 2 Department of Biomedical Engineering, Emory University and The Georgia Institute of Technology, 1841 Clifton Road NE, Atlanta, GA 30329, USA; 3 Institute for Applied Microelectronics (IUMA), University of Las Palmas de Gran Canaria (ULPGC), 35017 Las Palmas de Gran Canaria, Spain; sortega@iuma.ulpgc.es; 4 Advanced Imaging Research Center, University of Texas Southwestern Medical Center, 5323 Harry Hine Blvd, Dallas, TX 75390, USA; 5 Department of Radiology, University of Texas Southwestern Medical Center, 5323 Harry Hine Blvd, Dallas, TX 75390, USA

**Keywords:** hyperspectral imaging, clinical diagnosis, biomedical optical imaging, cancer, medical diagnostic imaging, artificial intelligence, machine learning

## Abstract

In contrast to conventional optical imaging modalities, hyperspectral imaging (HSI) is able to capture much more information from a certain scene, both within and beyond the visual spectral range (from 400 to 700 nm). This imaging modality is based on the principle that each material provides different responses to light reflection, absorption, and scattering across the electromagnetic spectrum. Due to these properties, it is possible to differentiate and identify the different materials/substances presented in a certain scene by their spectral signature. Over the last two decades, HSI has demonstrated potential to become a powerful tool to study and identify several diseases in the medical field, being a non-contact, non-ionizing, and a label-free imaging modality. In this review, the use of HSI as an imaging tool for the analysis and detection of cancer is presented. The basic concepts related to this technology are detailed. The most relevant, state-of-the-art studies that can be found in the literature using HSI for cancer analysis are presented and summarized, both in-vivo and ex-vivo. Lastly, we discuss the current limitations of this technology in the field of cancer detection, together with some insights into possible future steps in the improvement of this technology.

## 1. Introduction

Hyperspectral imaging (HSI), also referred to as imaging spectroscopy, integrates conventional imaging and spectroscopy methods to obtain both spatial and spectral information of a scene [1]. Unlike conventional RGB (red, green and blue) imaging, which only captures three diffuse Gaussian spectral bands in the visible spectrum (e.g., 380–740 nm), HSI increases the amount of data acquired beyond the capabilities of the human eye [2]. Hyperspectral (HS) sensors measure the aggregate signal of reflected, absorbed and emitted radiance at specific wavelengths of the material that is being observed. These sensors are capable of capturing a very large number of contiguous spectral bands (also called *spectral wavelengths* or *spectral channels*) across the electromagnetic spectrum (ES). Each pixel of an HS image, or average over a region of interest, has associated a specific vector of radiance values, commonly called *spectral signature* [1]. Image processing algorithms make use of these spectral signatures to automatically differentiate the materials observed by the sensor at each pixel [3]. These methods rely on the basis that different molecular compositions of each material present in nature have different responses to the incident light [1].

HSI is a promising non-invasive and non-ionizing technique that supports rapid acquisition and analysis of diagnostic information in several fields, such as remote sensing [4,5], archeology [6,7], drug identification [8,9], forensics [10,11,12], defense and security [13,14], agriculture [15,16], food safety inspection and control [17,18,19], among many others. Particularly, several studies can be found in the literature where HSI is applied to different medical applications [20,21,22]. It has been proven that the interaction between the electromagnetic radiation and matter carries useful information for medical diagnostic purposes [20]. As an alternative to other existing technologies for diagnosis, one of the strengths offered by HSI is being a completely non-invasive, non-contact, non-ionizing and label-free sensing technique. In medical applications, this technology has been employed in several different areas such as blood vessel visualization enhancement [23,24], intestinal ischemia identification [25], oximetry of the retina [26,27,28], estimation of the cholesterol levels [29], chronic Cholecystitis detection [30], histopathological tissue analysis [31,32,33,34], diabetic foot [35], etc. In recent years, medical HSI has started to achieve promising results with respect to cancer detection through the utilization of cutting-edge machine learning algorithms and increased modern computational power [20,21,36].

In this review, the basis of the different HS acquisition systems is explained, as well as their applications to the different tissue sample analysis, followed by a brief presentation of the most common data processing approaches employed to process HS information. In addition, a brief introduction to the optical properties of cancer tissues and the current investigations performed in the literature to study and analyze in-vivo and ex-vivo cancer tissue samples using HSI are presented.

## 2. Hyperspectral Imaging

### 2.1. Hyperspectral Image Acquisition Systems

**Figure 1:**
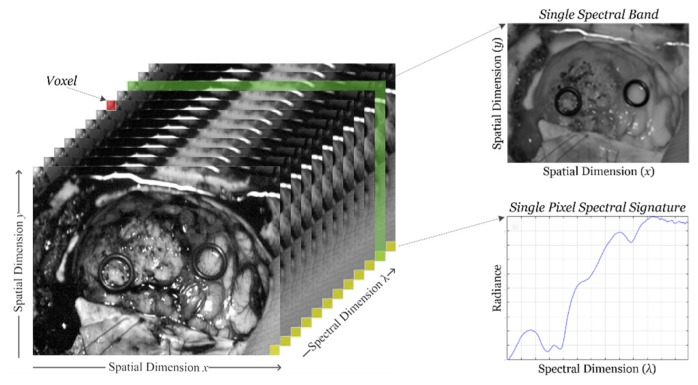
Hyperspectral imaging data. Basic structure of a hyperspectral imaging (HSI) cube, single band representation at a certain wavelength and spectral signature of a single pixel.

HSI sensors generate a three-dimensional (3D) data structure, called *HS cube*, where the spatial information is contained in the first two dimensions, while the third dimension encompasses the spectral information. Figure 1 shows the information structure of an HS cube. On one hand, each pixel of the HS image contains a full spectral signature of length equal to the number of spectral bands of the HS cube. On the other hand, a gray scale image of the captured scene can be obtained using any of the spectral bands that displays the spatial information provided by the image sensor at a particular wavelength.

**Figure 2:**
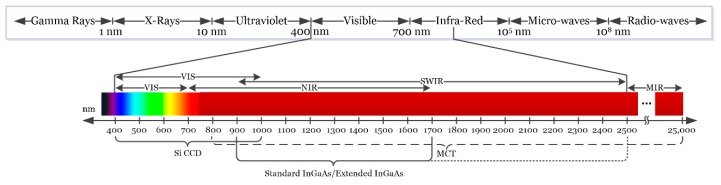
Electromagnetic spectrum. HSI is commonly employed between the visible and the medium-infrared range.

Depending on the type of sensor employed, HSI cameras will cover different spectral ranges. Figure 2 shows the partitioning of the entire electromagnetic spectrum (ES) and the range where HS images are commonly captured depending on the sensor type. Charge-coupled device (CCD) silicon-based sensors are sensitive in the visible and very near-infrared (VNIR) spectrum in the range between 400 and 1000 nm. The standard indium gallium arsenide (InGaAs) sensors are able to capture HS images in the near-infrared (NIR) range, between 900 and 1700 nm, extending in some cases the upper range to 2500 nm. Other types of sensors can reach larger spectral ranges. For example, the mercury cadmium telluride (MCT) sensors are able to acquire HS images in the short-wavelength infrared (SWIR) range, from 1000 to 2500 nm, being able also to reach 25,000 nm in some specific systems [37].

**Figure 3:**
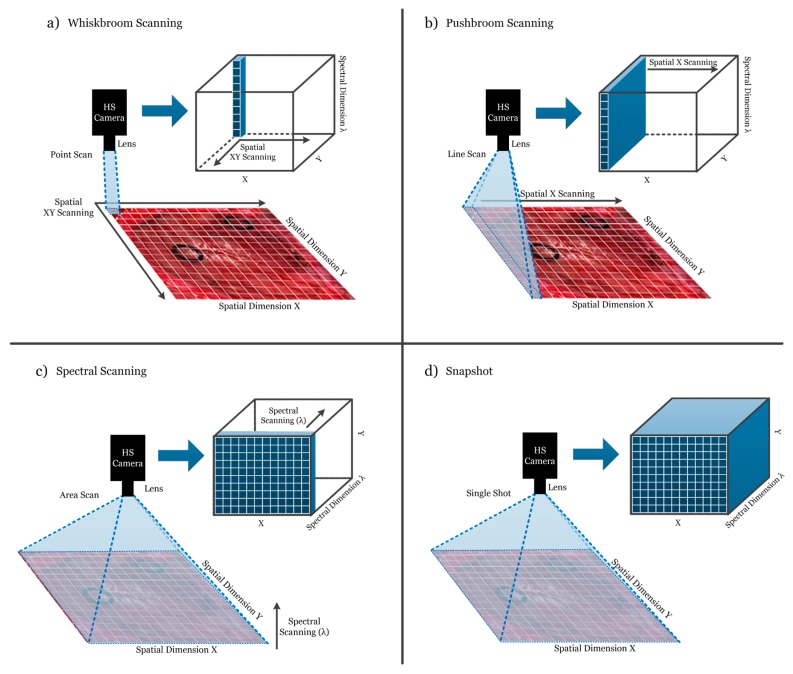
Hyperspectral camera types and their respective acquisition and data storage methods. (**a**) Whiskbroom camera; (**b**) Pushbroom camera; (**c**) Hyperspectral (HS) camera based on spectral scanning; (**d**) Snapshot camera.

HS cameras are mainly classified into four different types (Figure 3) depending on the methods employed to obtain the HS cube: *whiskbroom* (point-scanning) cameras, *pushbroom* (line-scanning) cameras, cameras based on spectral scanning (area-scanning or plane-scanning), and *snapshot* (single shot) cameras [17].

Whiskbroom cameras (Figure 3a) are characterized by capturing one single pixel at one time containing all its spectral information. The rest of the pixels of the scene are captured by scanning both spatial dimensions (*x* and *y*). Whiskbroom cameras have the main disadvantage of being very time-consuming during the image acquisition. However, they can achieve very high spectral resolutions, allowing researchers to perform a more detailed analysis of the spectral signature of the pixel that is captured.

Closely related to the previous camera type, pushbroom cameras (Figure 3b) offer a faster scanning solution compared to whiskbroom, but also obtain high spectral resolution. In this case, the camera captures one line of pixels of the scene (*y-axis*) at one time. The other spatial dimension (*x*-*axis*) is obtained by moving the field-of-view (FOV) of the camera in that direction. Pushbroom cameras are the most common systems in remote sensing field and other industrial sectors due to their high spectral resolution, showing reduced capturing times with respect to the whiskbroom cameras. The main disadvantage of spatial scanning cameras is the presence of spatial aliasing if movement artifacts occur during the acquisition period [37].

On the other hand, HS cameras based on spectral scanning (Figure 3c) are able to obtain the entire spatial information (*x-axis* and *y-axis*) of the scene for a certain wavelength at one time, performing a scanning in the spectral dimension (*λ-axis*). These cameras can achieve high spatial resolutions and fast acquisition times; however, the spectral resolution is typically lower when compared to the spatial scanning (whiskbroom and pushbroom) cameras. One of the main disadvantages of spectral scanning cameras is that they are not suitable for capturing moving objects due to the time required to perform the spectral scanning, which would induce spectral aliasing artifacts. LCTF (Liquid Crystal Tunable Filter) and AOTF (Acousto-Optic Tunable Filter) HS cameras employ optical filters electronically controlled to filter each wavelength and generate the complete HS cube by performing a spectral scanning. 

Finally, there is an emerging type of HS camera that can provide hyperspectral video, having the lowest acquisition time and allowing the acquisition of moving objects without performing any spatial or spectral scanning. Snapshot cameras (Figure 3d) capture the entire scene in a single shot that contains both the spectral and spatial information [38]. The main disadvantage of snapshot cameras is that the spectral and spatial resolutions are much lower with respect to the other camera types.

In summary, the order of spectral resolution from highest to lowest is line scanning and point scanning being about equal, followed by spectral scanning, and snapshot. The order of spatial resolution from highest to lowest is spectral scanning, line scanning and point scanning being about equal, and snapshot. The order of fastest to slowest acquisition times is snapshot, spectral scanning, line scanning, and point scanning. The use of high spectral resolution HS cameras for cancer analysis allows researchers to study in detail the optical properties of the tissues, identifying the most relevant spectral channels that are involved in a certain application. On the other hand, the use of high spatial resolution HS cameras permits the analysis of samples by combining the spectral and the morphological properties of the tissue. Finally, HS snapshot cameras are the most suitable option for real-time analysis situations, mainly when the analysis of in-vivo tissue is performed. In conclusion, the HS sensor type and acquisition system selected are highly application dependent. 

### 2.2. Hyperspectral Image Processing Algorithms

An extensive literature is available on the classification of HS images [39]. Traditionally, HSI has been widely employed in the remote sensing field, so the majority of algorithms developed to classify HS images are related to this field [40]. However, more recently, HSI is progressively being used in other fields, such as drug analysis [41,42], food quality inspection [17,18,19,43], automated clinical microbiology for pathogen identification directly from bacterial colonies [44,45,46], or defense and security [13,47], among many others. That is why the algorithms that were developed targeting remote sensing application have been adapted to classify different types of scenes.

Pixel-wise classification methods assume that each pixel is pure or a mix of pure pixels and can be assigned to a certain material based on its spectral information [48,49,50]. Pixel-wise classification algorithms can be divided into two types: *supervised* classifiers and *unsupervised* classifiers (also called *clustering* or *segmentation* algorithms). Furthermore, in recent years, the use of deep learning (DL) approaches to classify HS data has become increasingly common, achieving excellent results when compared with traditional machine learning (ML) algorithms [51]. When applied to medical HSI data, these algorithms face two main challenges: the high dimensionality and the limited number of samples. However, these challenges are not necessarily present in other HSI domains, but are more prevalent in medical HSI because of substantial inter-patient spectral variability.

The basis of supervised classification algorithms relies on training an algorithm on a set of spectral signatures with known class labels and using this trained model to assign new labels to unknown spectral signatures in a HS image. The training process of supervised algorithms must be performed with a library of spectral signatures where each type of signature has been identified with a certain membership class, with the goal that this library is sufficiently representative for generalization purposes. Moreover, regression-based, statistical ML methods based on linear discriminant analysis, decision trees, random forest (RF) [52,53], artificial neural networks (ANNs) [54,55,56], and kernel-based methods have been widely used to classify HS images. In particular, there are several types of kernel-based regression methods in the literature [57], where the support vector machine (SVM) classifier is the most commonly used algorithm. In the HSI field, SVMs provide good performance for classifying this type of data when a limited number of training samples is available [57]. Due to its strong theoretical foundations, good generalization capabilities, low sensitivity to the problem of dimensionality and the ability to find optimal solutions, SVMs are usually selected by many researchers over other traditional, regression-based ML classification paradigms for classifying HS images [20]. As a relevant example, a variant of the SVM classifier, called fuzzy SVM classifier, was employed in the development of an emotion recognition system based on facial expression images [58]. In the medical field, SVMs have been used to detect multiple sclerosis in subjects, employing stationary wavelet entropy to extract features from magnetic resonance images used as inputs of the SVM classifier [59]. Furthermore, the same technique combined with a directed acyclic graph method has been used to diagnose unilateral hearing loss in structural MRI [60], demonstrating that the SVM algorithm is a reliable candidate to work with a variety of medical image modalities. Other relevant algorithms employed to work in classification problems of high dimensional data in computational biology are the partial regression methods such as partial least squares (PLS) [61], partial least squares-discriminant analysis (PLSDA) [62] or sparse partial least squares-discriminant analysis (sPLSDA) [63]. PLS algorithms are suitable to work as a multivariate linear regression method that can deal with a large number of predictors, small sample size, and high collinearity among predictors [63], performing competitively with other state-of-the-art classification methods such as SVMs [64]. 

On the other hand, the goal of the unsupervised classifiers is to divide an image into a certain number of similar groups (also called *clusters*), where each group shares approximately the same spectral information and provides the correspondent cluster centroid [65,66]. Each cluster centroid represents a spectrum corresponding to a material in the scene, while the membership functions provide the weights for these spectra. Unlike the supervised classifiers, unsupervised methods do not require a training process using labeled samples. For that reason, they cannot provide the identification of the class that each pixel belongs to, only relative clustering with no information about the material’s nature. Although unsupervised clustering does not provide any discriminant features by itself, it could be used to delineate the boundaries of the different spectral regions presented in an HS image. Unsupervised algorithms, such as the K-means algorithm [67,68] and the Iterative Self-Organizing Data Analysis (ISODATA) technique [69,70,71], are the most common clustering algorithms employed in the literature using HS data [39]. Hierarchical clustering is an unsupervised method of cluster analysis that seeks to obtain a hierarchy of clusters [72,73]. Several hierarchical clustering algorithms have been employed to classify HS images, such as Hierarchical rank-2 non-Negative Matrix Factorization (H2NMF) [74], Hierarchical K-Means (HKM) [74,75], and Hierarchical Spherical K-Means (HSKM) [76]. Some works based on HS analysis for medical applications use unsupervised clustering as part of the classification algorithms, such as those for colon tissue cell classification [77] or laryngeal cancer detection [78].

Deep learning (DL) techniques have been used for many applications of remote sensing data analysis, such as image processing, pixel-wise classification, target detection, and high-level semantic feature extraction and scene understanding [51]. Computationally, DL generates predictive models that are formed by several stacked processing layers with ‘neurons’ that can activate with learned weights to discriminate different representations of data with multiple levels of abstraction. DL architectures can extract intricate features in large datasets through an iterative, error backpropagation approach that determines the gradients that lead to successful changes of internal parameters [79]. While conventional machine learning techniques are limited in their ability to process data in its input form, DL methods can learn new mathematical representations from the input data required for detection or classification. These multiple levels of representation are obtained by non-linear modules that modify the representation at one level (starting with the raw input) into a representation at a higher, slightly more abstract level, where very complex functions can be learned [79]. 

Many DL frameworks have been applied to HS images in the literature. Deep belief networks (DBNs) [80] and convolutional neural networks (CNNs) [81,82] have been employed to process and classify HS remote sensing data, improving upon the results obtained with conventional SVM-based algorithms [83,84,85,86]. CNNs have also been employed to extract high-level spatial features from HS data in a spectral–spatial feature extraction algorithm for HS image classification [87]. In the medical field, DL is emerging in recent years as a powerful tool in the field of translational bioinformatics, imaging, pervasive sensing, and medical informatics [88]. As an example, deep neural networks (DNNs) and CNNs have been employed to classify electrocardiogram signals [89,90,91], detect retinal vessels [92,93,94,95], classify colorectal polyps [96,97,98], and cancer analysis [99,100,101,102,103]. On the other hand, the use of DL techniques in medical HSI is recent because of the large amounts of training data required, and currently there are not many medical HSI databases available. 

## 3. Cancer Optical Properties

**Figure 4:**
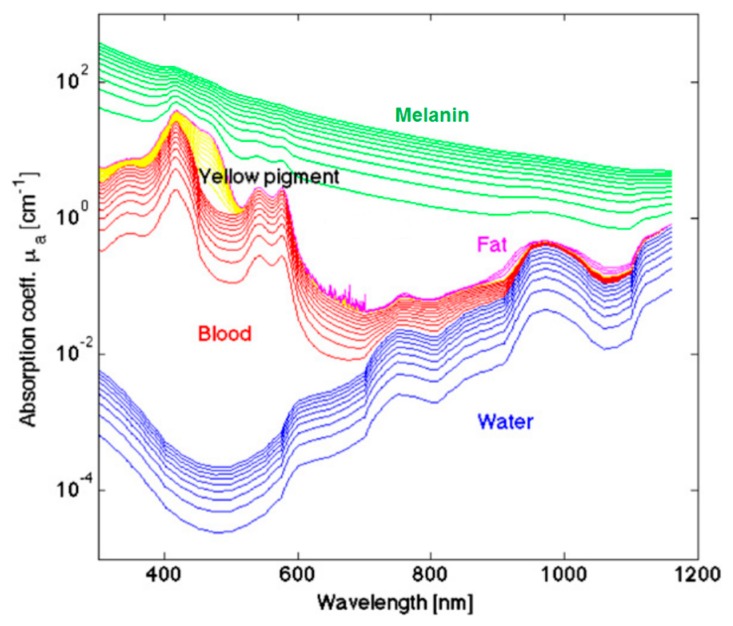
A few representative major molecular contributions to the absorbance at wavelengths of light typical for HSI investigations of biological tissue [107]. Reproduced with permission from [107]; published by IOP Publishing (2013).

The measured optical spectra of biological tissues from 400 to 1000 nm cover the visible and NIR regions and can be broken down at the molecular level, which greatly contributes to the reflectance values measured in certain ranges. The hemoglobin (Hb) absorption and reflectance spectra vary substantially between the oxygenated and deoxygenated states, and Hb is a major spectral contribution of biological tissue in the range of 450 to 600 nm [104]. Deoxygenated Hb shows a single absorbance peak around 560 nm, while oxygenated Hb shows two equal absorbance peaks around 540 and 580 nm [105]. The region of the NIR spectrum from 700 to 900 nm corresponds with the scattering dominant optical properties of collagen [106]. The NIR region is referred to as the scattering dominant region for biological tissues, mainly composed of fat, lipids, collagen, and water. The molecular contributions of absorbance at wavelengths in the typical HSI range are shown in Figure 4. The relationship of absorbance is inverse to reflectance measured by HSI systems. For a more detailed summary of the optical properties of biological tissue, the interested reader is directed to the canonical review by Jacques [107]. 

Deal et al. investigated the contributions of normal and neoplastic colonic autofluorescence, which is the reflectance observed from the endogenous fluorescent molecules in biological tissue, using excitation wavelengths from 360 to 550 nm and emission at 555 nm, separated with a long-pass emission filter and dichroic beamsplitter [108]. Investigating neoplastic tissues, colorectal adenocarcinoma and adenomatous polyps, demonstrated that some autofluorescent molecules, such as elastin and nicotinamide adenine dinucleotide (NADH), had a significantly different abundance compared to normal colonic tissues, while other molecules, such as collagen, flavin adenine dinucleotide (FAD), and protoporphyrin IX (PPIX), showed no change between normal and neoplastic tissues. However, the authors acknowledge the limitation of performing their experiments with pairs of healthy and cancer from only nine patients and that non-neoplastic normal tissue from a diseased colon may vary molecularly from normal healthy colon tissue in a subject devoid pathology.

Monte-Carlo (MC) methods applied to medical HSI use simulations of photons with random parameter perturbations to simulate the interaction of light and biological tissues. Hermann et al. developed an MC simulation of HS illumination in the visible and NIR regions to study reflectance signals of a multi-layer model in silico with different blood volume and oxygen saturations per layer [109]. The authors confirmed the absorbance peaks of water at 1000 nm and reflectance ratios at 580/800 nm for Hb. Interestingly, the authors also contend that blood volume fractions of 5% and 10% are detectable at depths of up to 1 mm in the simulated biological tissue, but changes beyond this depth are likely not resolvable. Additionally, it is possible to correlate in vivo measured reflectance values from various cancers and healthy tissues to extract meaningful optical tissue properties associated with distinct molecular components, such as collagen, keratin, and Hb, using an inverse-MC approach, which has been previously reviewed for its application to cancer diagnosis [110].

## 4. Medical Hyperspectral Imaging for Cancer Analysis

In the previous sections, the basis of the HSI technology, the main algorithms employed to process this type of data and the optical properties of cancer tissue have been described. This section is devoted to detail the state-of-the-art methods and primary research on the use of HSI within the medical field, focusing on cancer analysis. 

**Figure 5:**
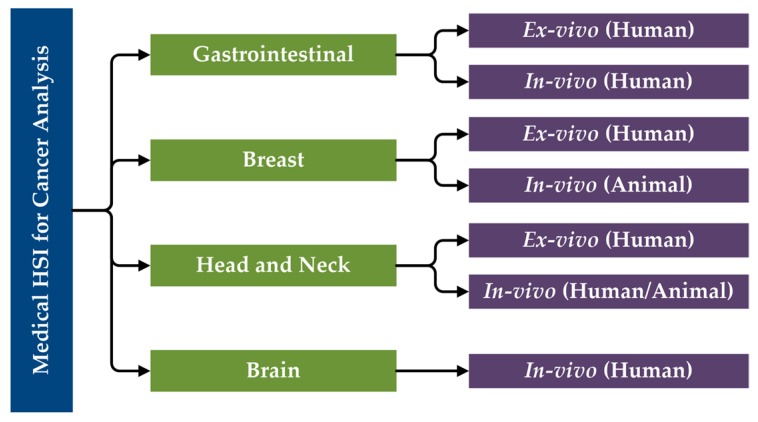
Taxonomy of the state-of-the-art methods of medical HSI for cancer detection that are reviewed in this paper, organized by organ systems.

The primary research performed in the literature related to the use of HSI for cancer analysis can be categorized first by organ systems, next the type of tissue samples and experimental design (ex vivo, in vivo), and finally the type of subjects (human, animal). Moreover, in vitro studies will not be included in this review of state-of-the-art works for cancer detection. Considering this, a specific taxonomy has been established related to the studies performed in this area that have been reviewed in this paper (Figure 5). 

While it is possible to detail the investigations of HSI for cancer analysis altogether, HSI systems are not standardized (see Section 2.1), as different technologies were used in the following studies. As will be summarized in Table 1, most of the studies work in the VNIR spectral region, employing CCD sensors. However, in some studies, the NIR region is also explored, requiring the use of InGaAs sensors. Halogen or xenon lamps are generally used as illumination systems for HSI applications, and sometimes optical fibers are used for light transmission to avoid the high temperatures produced by these types of light sources or to concentrate the light into a certain area. The main characteristics of the systems employed in each study presented in this literature review will be detailed in the summary table shown at the end of this section (Table 1), as well as a summary of the main characteristics of them, sorted by the year of publication. 

### 4.1. Gastrointestinal Cancer

#### 4.1.1. Clinical Need for HSI of Gastrointestinal Cancer

There are strong indications for endoscopy of the upper digestive tract and colonoscopy of the bowel for early detection of digestive tract cancers, with regular screenings for higher risk individuals [111]. Gastric carcinomas, most frequently caused by infection with helicobacter pylori or dietary contributions, are the second leading cause of cancer death globally. For the approximately two-thirds of gastric cancer patients that are diagnosed with locally advanced or metastatic disease, with a five-year survival of only 10%, the only curative therapy remains surgical resection [112]. Colorectal cancers, mostly adenocarcinomas, are thought to arise mainly from secondary risk factors of dietary and lifestyle origins, such as excessive caloric and fat intake, smoking, and physical inactivity [113]. Laparoscopic surgeries, performed in a way that is minimally invasive and guided by endoscopy, have been shown to be clinically equivalent in randomized controlled trials [114]. However, during this minimally invasive surgery, there is a loss of tactile feedback that surgeons often require, so there is a need to overcome this lost information [115]. HS imaging has been proposed as a solution to this problem with potential for more accurate digestive tract cancer resections.

#### 4.1.2. Ex-Vivo Human Gastric Cancer

**Figure 6:**
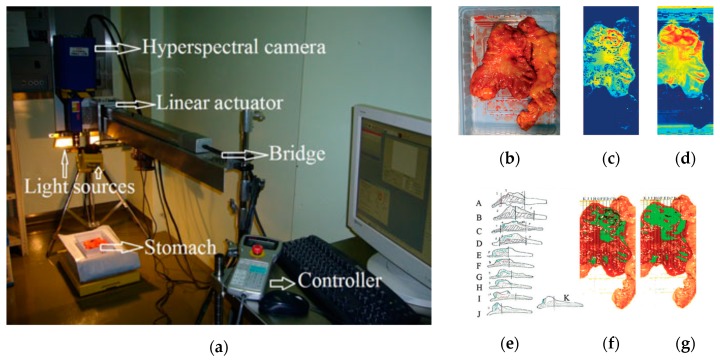
Gastric cancer detection acquisition system, cancer detection results using the NDCI and integral filter, and comparison with histopathological results obtained in [116]. (**a**) HS acquisition system setup; (**b**) RGB representation of the ex-vivo sample; (**c**) Cancer enhanced regions using an integral filter in the hyperspectral image (1057–2440 nm); the tissues are shown in a blue to red spectrum, where the red regions represent the tumor; (**d**) Cancer enhanced regions using NDCI; (**e**) Pathological sectioning and results; (**f**) Detected tumor using an integral filter; (**g**) Detected tumor using NDCI. Reproduced with permission from [116]; published by Wiley (2011).

In 2011, Akbari et al. performed a study to identify gastric tumors in human ex-vivo tissues using an HS system, which was capable of capturing images in the range between 1000 and 2500 nm, obtaining 239 spectral bands [116]. An integral filter and the normalized cancer index (NDCI) was applied to perform an automatic classification of the tumor tissue determining the boundaries between tumor and normal tissue using histopathological analysis to validate their results (Figure 6). From their experiments, they determined that the spectral regions between 1226 and 1251 nm and 1288 and 1370 nm are the most salient ranges for distinguishing between non-cancerous and cancerous gastric tissue. 

In 2013, Kiyotoki et al. collected HS images in the spectral range comprised between 400 and 800 nm from ex-vivo tissue gastric samples to perform a preliminary study of gastroduodenal tumors removed by endoscopic resection or surgery from 14 different patients [117]. The system was able to obtain HS images comprised of 72 spectral bands with a spatial dimension of 640 × 480 pixels. Using these images, they were able to determine the optimal wavelength that allowed the most accurate classification between tumor and normal mucosa using the cutoff point method at the 726 nm wavelength. The sensitivity, specificity, and accuracy obtained in the test samples were 78.8%, 92.5% and 85.6%, respectively. This work was expanded upon in 2015 by the same group, increasing the number of patients to 96 and performing the selection of the optimal wavelength using the Mahalanobis distance, which in this case was 770 nm [118]. Sensitivity, specificity, and accuracy results obtained were 71%, 98%, and 85%, respectively, demonstrating that the increment in the number of patients to analyze did not decrease the accuracy of the method. Although the classification method employed to distinguish the different types of samples was quite basic, the studies revealed promising results in the use of HSI as a diagnostic tool for gastric cancer.

Baltussen et al. performed a study of laparoscopic HS imaging using two HS cameras collectively sensing between 400 and 1700 nm to distinguish normal fatty tissue, healthy colorectal mucosa, and adenocarcinoma in order to provide more diagnostic information back to surgeons, given the loss of tactile feedback during endoscopic procedures [119]. The authors utilized 32 patient samples to perform a three-class detection using quadratic SVMs of fat, muscle, and tumor, and obtained a tissue-level accuracy of 88% and a patient-level accuracy of 93%. One limitation of the presented HSI technique was focusing only on muscle and fat as normal tissues, but the authors acknowledge that future work should involve the entire specimen.

#### 4.1.3. In-Vivo Human Colon Cancer

HS endoscopic systems have been used to study in-vivo colorectal tumors in the literature. One of the main studies in this field was performed in 2016, when Han et al. used a flexible hyperspectral colonoscopy system based on a motorized filter wheel, capable of obtaining 27 different bands in the range comprised between 405 and 665 nm, to discriminate between malignant colorectal tumors and normal colonic mucosa in human patients [120]. They used a wavelength selection algorithm based on the recursive divergence method to identify the most relevant wavelengths in the spectral range employed, demonstrating that HSI can be used in-vivo for outlining the disease region and enhancing the microvascular network on the mucosa surface. The sensitivity and specificity results achieved in this study reach up to 96% and 91%, respectively, using all the available bands. On the other hand, the lower results obtained using only one spectral channel were 94% and 82% of sensitivity and specificity.

### 4.2. Breast Cancer

#### 4.2.1. Clinical Need for HSI of Breast Cancer

Breast conserving surgery, also known as lumpectomy, with adjuvant radiation therapy, is the recommended surgical approach over traditional mastectomy without radiation for women diagnosed with early breast cancer. Women diagnosed with stage I or II breast cancer showed increased overall survival and disease-survival rates when treated with lumpectomy and radiation compared to complete mastectomy without radiation therapy, and this result was seen for all age groups and cancer types [121]. Successful breast conserving surgery is directly dependent on complete removal of the tumor mass with adequate margins, meaning there is a buffer of healthy tissue on the free cut edge. There is evidence to suggest that conservatively around 20% of women who undergo partial mastectomy have a final, post-operative diagnosis of positive margin status, which requires additional surgeries, with some studies reporting higher figures [122]. Intraoperative biopsies along with pathologist consultations are necessary tools to guide surgeons, but the need remains to provide more intraoperative diagnostic information with one potential solution being HSI.

#### 4.2.2. In-Vivo Animal Breast Cancer

One of the first and most relevant works performed using HSI to study breast cancer was performed in 2007 by Panasyuk et al. [123]. In this work, a HS system based on LCTFs was used to acquire HS images in the visual spectral range, between 450 and 700 nm and composed of 34 bands, during intraoperative surgery of 60 rats affected by an induced breast cancer. They generated classification maps, where different types of tissue including tumor, blood vessels, muscle, and connective tissue were clearly identified. Furthermore, comparison to the histopathological examination of the tumor bed yielded a sensitivity of 89% and a specificity of 94% for the detection of the residual tumor by HS imaging. One of the limitations of this work was the use of light-emitting diode (LED) illumination in the HS acquisition system, which produced a non-standard spectral signature because LED light does not provide a uniform, broadband spectrum, such as that obtained by halogen or xenon light sources.

McCormack et al. performed a study of mouse models of breast cancer that aimed to evaluate the use of in-vivo HSI for microvessel oxygen saturation (sO2) monitoring during surgical procedures, studying also the response of the microvessels to different types of treatments [124]. The HS acquisition system was based on LCTFs and a halogen lamp, capturing images in the spectral range between 500 and 600 nm and composed of 26 bands because the absorption levels of both oxy and deoxy-hemoglobin are known to peak in this range. 

#### 4.2.3. Ex-Vivo Human Breast Cancer

Breast cancer has also been studied using ex-vivo samples with the goal of automatically delineating the regions of interest (ROI) in the samples and classifying the tumor and normal tissue samples. In 2013, two studies were published with both previously mentioned goals using an HS system capable of obtaining images in the spectral range between 380 and 780 nm. The study conducted by Kim et al. performed an automatic ROI detection based on contrast and texture information achieving a true positive rate (TPR) and a true negative rate (TNR) of 97.3% and 95.9%, respectively, similar to the results obtained in a manual segmentation (98.7% and 96.4%) [125]. In the study performed by Pourreza-Shahri et al., the authors performed a feature extraction (using the Fourier coefficient selection features method) and a dimensional reduction (using the Minimum Redundancy Maximum Relevance method) to the HS images and then performed an automatic classification, using the SVM classifier with the radial basis function (RBF) kernel, of the tissue samples, differentiating between cancerous and non-cancerous tissue [126]. Sensitivity and specificity results of 98% and 99%, respectively, were obtained, demonstrating that HSI is a powerful imaging modality that has potential for use in the aided diagnosis of breast cancer. 

### 4.3. Head and Neck Cancer

#### 4.3.1. Clinical Need for HSI of Head and Neck Cancer

Head and neck (H&N) cancers are the sixth most common cancer worldwide [127]. Approximately 90% of the cancers at origin sites of the upper aerodigestive tract, which includes the oral cavity, nasal cavity, pharynx, and larynx, are squamous cell carcinoma (SCC). There are several well studied risk factors for H&N SCC, including consumption of tobacco and alcohol and oral infection with human papilloma virus (HPV) [128]. Patients with SCC typically present it at an advanced stage (stage 3 or 4 disease) [129]. The mainstay treatment for SCC is surgical cancer resection. The single largest predictor of patient outcomes for SCC resection is the successful removal of the entire SCC from the surgical wound bed, referred to as negative margins. The presence of positive or close (less than 2 mm of tissue clearance) margins after surgery greatly increases the likelihood for locoregional disease recurrence and additional surgeries [130]. Surgeons rely on intra-operative pathologist consultations with the surgical pathology department to ensure that negative margins are obtained through the use of frozen-section (FS) microscopic analysis of the resected specimens. Despite this, in the literature it is reported that up to 20% of patients will have a final diagnosis of positive or close margins despite having negative FS intraoperatively. Because of the difficulties in treating this challenging form of cancer, the estimated five-year survival rate of SCC is only 40 to 60% with treatment [131]. There exists a great need to provide more near-real-time information and guidance to the operating surgeon.

#### 4.3.2. In-Vivo Animal Head and Neck Cancer

Head and neck SCC was studied in-vivo using HS images from mice with SCC. The studies were performed in the VNIR range between 450 and 950 nm using a CRI system (Perkin Elmer Inc., Waltham, Massachusetts), which is comprised of a Xenon illumination source, LCTF, and a 16-bit CCD camera capturing images at a resolution of 1040 by 1392 pixels and a spatial resolution of 25 µm per pixel. Lu et al. published several works in this field, where the tensor decomposition, PCA and KNN methods were employed to perform a feature extraction and automatic classification, achieving a sensitivity of 93.7% and a specificity of 91.3% in the discrimination of tumor and normal tissue [132,133]. Furthermore, the group also studied the tumor margin during the surgical procedures performing an in-vivo/in-vitro registration between the in-vivo HS images and the histological images to validate the results [134]. 

**Figure 7:**
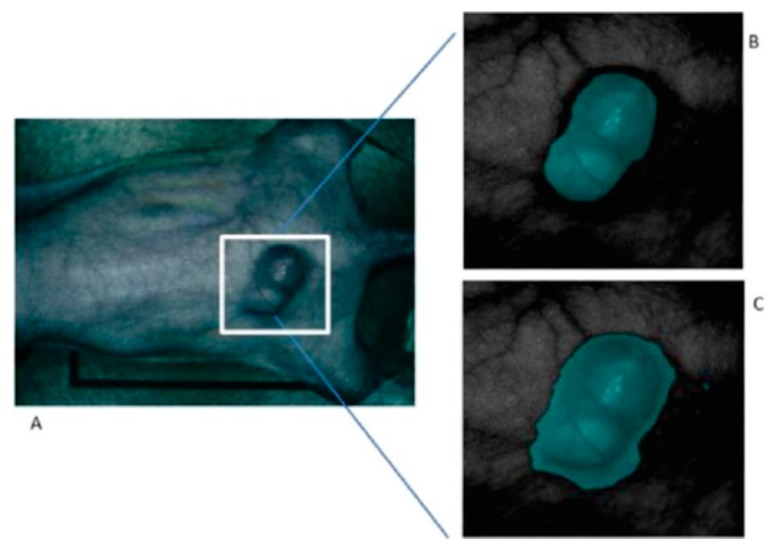
Result of the tumor identification using the Minimum-Spanning Forest method developed in [137]. (**A**) Synthetic RGB image of the original mouse; (**B**) Corresponding gold standard image; (**C**) Classification result obtained. Reproduced with permission from [137]; published by IEEE (2015).

On the other hand, their research has analyzed which pre-processing techniques are more suitable to compensate the variations of the environmental conditions during the acquisition inside an operating theatre [135,136]. In the work published in 2015, a method based on the mRMR (maximal Relevance and Minimal Redundancy) algorithm was proposed to address the problem of glare that usually appears in the HS images, improving the sensitivity and specificity results to 94.4% and 98.3%, respectively. In addition, other techniques were studied, such as the use of a minimum-spanning forest (MSF) algorithm for an automatic classification and segmentation of the in-vivo HS images [137]. Figure 7 shows the results from the MSF algorithm applied to an HS image of an in-vivo mouse xenografted with a line of human head and neck SCC, obtaining an accurate identification of the head and neck tumor with respect to the gold standard.

#### 4.3.3. Ex-Vivo Human Head and Neck Cancer

Active research into the application of HSI for H&N cancers is led by our group under Baowei Fei. Currently, all experiments explore H&N cancers including SCC and thyroid cancer in ex-vivo surgical tissue specimens and use the previously described CRI Maestro HS acquisition system in the VNIR spectral range, from 450 to 950 nm, with a spatial resolution of 1392 × 1040 pixels (25 µm per pixel), capturing 91 spectral bands.

**Figure 8:**
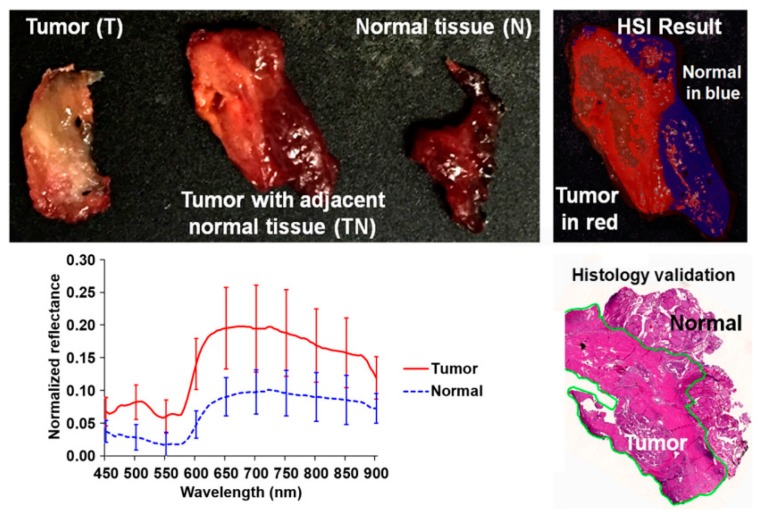
Preliminary results obtained in the tumor margin delineation for head and neck cancer [138]. After hyperspectral image acquisitions (top-left), the tissue was processed histologically, and tumor margins were outlined on the pathology image (bottom right) by a pathologist, which was used to validate the results of the classification (top-right). The average spectral curves are shown at the bottom left for each type of tissue, i.e., tumor, normal, and tumor with adjacent normal tissue. Reproduced from [138]; Creative Commons BY 4.0; published by SPIE (2017).

In 2017, several works were published in this area with the goal of discriminating cancerous and non-cancerous tissue. Fei et al. achieved an accurate delineation of the boundaries between the normal and cancerous tissue using head and neck ex-vivo samples compared with the histopathological results (Figure 8) [138]. The ensemble linear discriminant analysis (LDA) was employed to perform the classification, achieving an average accuracy, sensitivity and specificity of 90%, 89% and 91%, respectively, using oral cavity samples and an average accuracy, sensitivity and specificity of 94%, 94% and 95%, respectively, using thyroid samples. Autofluorescence, fluorescence with 2-deoxy-2-[(7-nitro-2,1,3-benzoxadiazol-4-yl)amino]-D-glucose (2-NBDG) and proflavine images were also classified and compared with the HSI results, demonstrating that HSI offered better results over the other alternative reflectance-based imaging modalities (an improvement of more than 7% of accuracy). 

In addition, Lu et al. increased the number of patients (N=36) and performed a comparison using different machine learning classification approaches, reinforcing the conclusion obtained from the other study, where the ensemble LDA outperformed other traditional machine learning algorithms [139]. In this study, both intra-patient and inter-patient classifications were performed, as well as different classifications using different spectral regions within the VNIR range (450–600 nm, 605–850 nm, 855–900 nm, and 450–900 nm). Finally, the authors contend that the use of the entire spectral range (from 450 to 900 nm) provides the best accuracy results.

Recently, one of the few studies performed in the HS literature regarding the use of deep learning methods to classify HS images with the goal of distinguishing cancerous and non-cancerous tissue was performed by Halicek et al. [140]. The authors developed a CNN classifier to process the ex-vivo tissues from 50 different patients and compared the deep learning method with traditional machine learning approaches, demonstrating that CNNs outperform the traditional classifiers in this case. 

Additionally, Halicek et al. implemented an inception-style CNN to investigate the ability of HSI to discriminate between cancer and normal tissues and obtained an AUC of 0.82 for SCC versus normal H&N tissue and 0.95 for thyroid carcinoma versus normal thyroid tissues. Also in this work, normal multi-class sub-classification was performed with an AUC of 0.94 for detection of normal squamous epithelium, mucosal gland, and skeletal muscle. For thyroid cancers, it was determined that benign thyroid hyperplasia can be detected from medullary or papillary thyroid carcinomas separately with above 0.91 AUC [141]. These preliminary results suggest that HSI has potential to be used in optical biopsies for H&N tissues to provide information beyond just binary cancer classification, but the study employed only 21 patient-excised surgical specimens.

#### 4.3.4. In-Vivo Human Aerodigestive Tract Cancer

**Figure 9:**
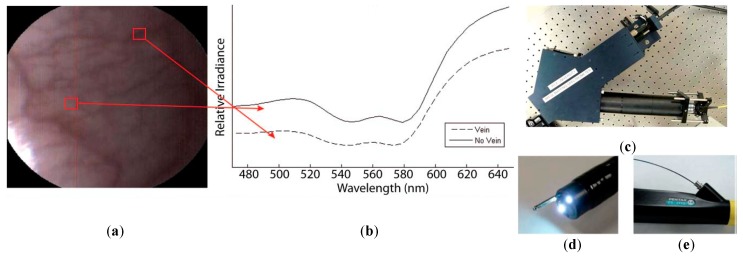
HS image example of the lower lip of a normal human acquired with the image mapping spectroscopy (IMS) endoscope developed in [142]. (**a**) RGB representation; (**b**) Spectral signature of the normal tissue pixel and a vein pixel; (**c**) Clinical setup of the IMS endoscope; (**d**) Miniature imaging end of the IMS endoscope; (**e**) Fiber optics of the IMS endoscope inserted into the instrument channel. Reproduced from [142]; Creative Commons BY 4.0; published by SPIE (2011).

A few studies can be found in the literature using HSI to analyze in-vivo samples of human subjects. Mainly, the studies are related to the use of endoscopic systems attached to an HS camera. In 2011, Kester et al. developed a customized real-time snapshot HSI endoscope system based on an image mapping technique and light dispersion that is capable of operating at frame rates of 5.2 fps (frames per second), obtaining HS cubes of 48 bands in the visible range between 450 and 650 nm, with a spatial resolution of 100 µm [142]. Using this system, they were able to capture in-vivo tissue, resolving a vasculature pattern of the lower lip while simultaneously detecting oxy-hemoglobin. Figure 9 shows an example of the spectral signatures obtained by the system and the developed acquisition system.

Another study was published by Jayanthi et al. related to the use of diffuse reflectance spectroscopy for early detection of malignant changes in the oral cavity [143]. The system was able to capture HS information within the visible spectral range (from 400 to 700 nm) based on a snapshot light dispersion technique, obtaining 40 different bands. They used PCA for dimensionality reduction and LDA for automatic classification of the data. They achieved sensitivity and specificity results higher than 95% in the discrimination between different lesions, such as normal/healthy, hyperplastic, dysplastic and SCC tissues.

In 2016, laryngeal cancer was investigated by Regeling et al. using a flexible endoscopy coupled to an HSI system that was able to obtain HS images composed of 30 bands in the visual spectrum between 390 and 680 nm [78]. This system was employed to obtain in-vivo HS images that required substantial image pre-processing, such as registration due to the patient’s heartbeat and noise removal due to specular reflections [144]. The images were registered using a rigid image-to-image registration based on normalized cross-correlation (NCC); the noise was reduced using the minimum noise fraction (MNF) transformation; and the glare was detected using a customized method. For classification, a random forest (RF) algorithm was applied to distinguish between healthy and cancerous tissues, achieving an overall accuracy of 88%.

Also in 2016, Laffers et al. employed a rigid HS endoscopic system to capture HS images between 390 and 680 nm of the oral cavity and oropharynx from 85 patients [145]. However, in this study they only took into consideration three patients, one of them used for training the algorithm and the other two for validation purposes. The classification results obtained using the RF algorithm were sensitivities of 61% and 43%, and specificity of 100% in the two validation patients. These reduced sensitivity values could be mainly produced by the low number of patients involved in the training of the classification algorithm, which would not correctly handle inter-patient variability for the training phase. 

**Figure 10:**
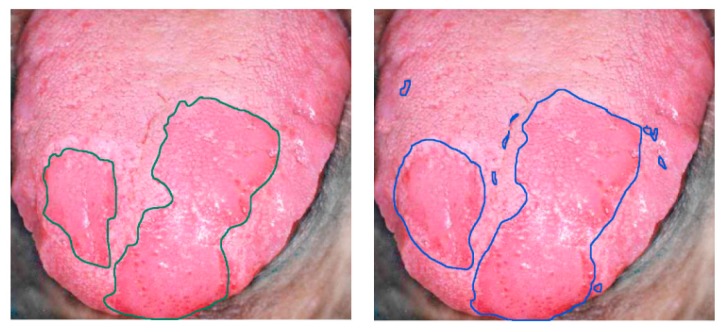
Delineation of the tongue tumor region in [146]. Expert labeling (**left**) and classifier prediction of tumor regions (**right**). Reproduced from [146]; Creative Commons BY 4.0; published by MDPI (2012).

Finally, tongue cancer of in-vivo human samples was studied in 2012 by Liu et al. using HSI [146] (Figure 10). The HS system utilized was based on an acousto-optic tunable filter (AOTF), capturing 81 bands in the VNIR spectral range comprised between 600 and 1000 nm. They developed a classifier based on the sparse representation (SR) method and compared the results obtained using traditional machine learning algorithms such as SVM and RVM (Relevance Vector Machine) classifiers. Sensitivity and specificity results of 91.3% and 93.7%, respectively, were obtained, increasing the accuracy by more than 4% with respect to the other two methods.

### 4.4. Brain Cancer

#### 4.4.1. Clinical Need for HSI of Brain Cancers

Brain tumors are categorized based on their histology and molecular parameters [147], being malignant gliomas the prevailing form of primary brain tumors in adults, causing between 2 and 3% of cancer deaths worldwide [148]. Surgery is one of the major treatment options for brain tumors alongside radiotherapy and chemotherapy [149]. Nevertheless, the surgeon’s naked eye is often unable to accurately distinguish between tumor and normal brain tissue as brain tumors infiltrate and diffuse into the surrounding normal brain. During neurosurgeries, it is frequent that too much normal brain tissue is taken out (called *safety margin*) or that tumor tissue is unintentionally left behind (called *residual tumor*). Several studies have demonstrated that the residual tumor is the most common cause of tumor recurrence and it is a major cause of morbidity and mortality [150,151,152]. In contrast, over-resection of brain tumor tissue has been shown to cause permanent neurological damages that affect patients’ quality of life [153]. 

Several image guidance tools, such as intraoperative neuronavigation, intraoperative magnetic resonance imaging (iMRI), intraoperative ultrasound (iUS) and fluorescent tumor markers (for example 5-aminolevulinic acid, 5-ALA), are commonly used to assist surgeons in the delineation of brain tumors. Conversely, these technologies present several limitations. Intraoperative neuronavigation is affected by the brain shift phenomenon [154], where the preoperative image link to the patient is affected by the brain deformation produced after craniotomy and durotomy. iMRI significantly extends the duration of the surgery (between 20 and 75 min per image [155]), generating a limited number of images [156] and requiring special operating rooms. On the other hand, iUS is inexpensive, real-time and unaffected by brain shift [157,158,159,160]. However, the use of iUS can cause the resection of histologically normal parenchyma [18]. Finally, although 5-ALA is able to identify the tumor boundaries, it produces relevant side effects on the patient and can only be used for high-grade tumors [161,162]. Thus, HSI can be a potential solution to the intraoperative margin delineation of brain tumors, being a label-free and non-ionizing imaging modality. 

#### 4.4.2. In-Vivo Human Brain Cancer

Some relevant studies with the goal of solving the problems exposed above, related to the intraoperative detection and delineation of brain tumors, can be found in the literature. In these studies, the development of machine/deep learning algorithms using HS images of in-vivo brain cancer has been investigated for the identification of the brain tumor margins [163,164,165,166,167,168,169,170,171,172,173,174,175].

All these studies were generated as outcomes of the European project HELICoiD (*HypErspectraL Imaging Cancer Detection*-618080) the main goal of which was to use HSI to generalize a methodology to discriminate between normal and malignant tissues in real time during neurosurgical procedures [173,176]. For this purpose, an intraoperative demonstrator was designed and built with the aim of acquiring intraoperative HS images and processing them in real time to assist neurosurgeons during resection [171]. This demonstrator was able to capture HS images in the spectral range comprised between 400 and 1700 nm in approximately 2 min using two pushbroom cameras. Two HS cubes were obtained, one in the VNIR range (400 to 1000 nm) formed by 826 spectral bands and a high spatial resolution of 1004 × 1787 pixels (129 × 230 mm) and another one in the NIR range (900 to 1700 nm) formed by 172 spectral bands and a low spatial resolution of 320 × 479 pixels (153 × 230 mm). However, they only used the information of the NIR range in two of the preliminary studies [170,177]. In 2015, Fabelo et al. employed labeled samples from 33 HS cubes of 22 different patients to study the use of three different supervised classifiers (SVMs, RF and ANNs) for the discrimination between tumor, normal and background (called *other*) samples, using an inter-patient 10-fold cross-validation method [173]. The results achieved were promising, showing specificity and sensitivity values higher than 96% for each class in both spectral ranges. In 2017, the same data were analyzed by Ravi et al. in [170], where 22 dimensional reduction approaches were evaluated and compared with a proposed new method called Fixed Reference T-distributed Stochastic Neighbors (FR-t-SNE). These methods were employed to generate three-band images from the HS cubes in order to provide high contrast images used as inputs of a semantic segmentation classifier. The results were evaluated using an inter-patient validation and a leave-one-out cross-validation method, achieving overall accuracy results higher than 80% and 71%, respectively. In addition, due to the high dimensionality of the VNIR data and the goal of the project to process the data in real time, a pre-processing chain able to obtain HS cubes of 129 spectral bands was proposed by Fabelo et al. in [169]. In this study, it was demonstrated the importance of the pre-processing of the HS images, providing even better results when the HS cubes were pre-processed.

**Figure 11:**
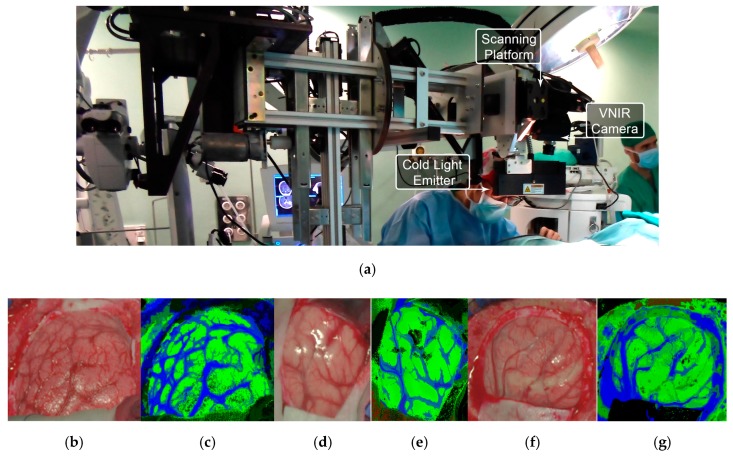
HELICoiD demonstrator [164] and normal brain image results obtained from the validation database employed in [171]. (**a**) HELICoiD demonstrator; (**b**,**d**,**f**) Synthetic RGB images; (**c**,**e**,**g**) Thematic maps of the HS image, where the normal tissue is represented in green color, the hypervascularized tissue in blue and the background in black. Reproduced from [171]; Creative Commons BY 4.0; published by MDPI (2018).

**Figure 12:**
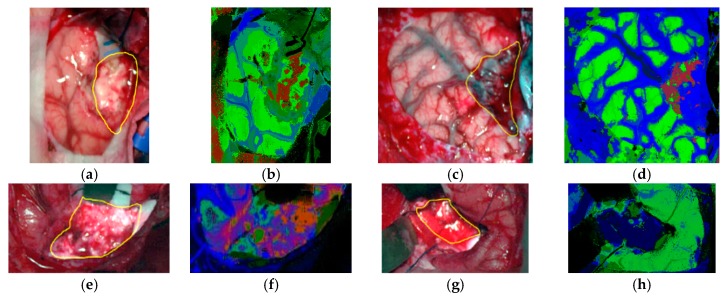
Tumor tissue identification results obtained from the validation database employing the HELICoiD demonstrator in [171]. (**a**,**c**,**e**,**g**) Synthetic RGB images; (**b**,**d**,**f**,**h**) Thematic maps of the HS image, where the tumor tissue is represented in red color, the normal tissue in green, the hypervascularized tissue in blue and the background in black. Reproduced from [171]; Creative Commons BY 4.0; published by MDPI (2018).

In subsequent studies, the same database was supplemented with more acquired data, and the labeling of the data was refined by using a customized semi-automatic labeling tool developed to this end, achieving more than 300,000 labeled pixels from 36 HS cubes that belong to 22 different patients [177]. Due to the difficulties of performing a reliable labeling of the NIR images, mainly because of the lack of high spatial resolution, only VNIR samples were labeled. This database is publicly available and was employed in some studies [177]. In 2018, Fabelo et al. presented in detail their classification algorithm for HS brain cancer detection, where the use of a supervised classification approach (based on a combination of a SVM, PCA and KNN filter algorithms) mixed with an unsupervised segmentation method (K-means), through a majority voting procedure, was able to achieve thematic maps identifying and delineating the tumor boundaries [164]. The results were quantitatively and qualitatively evaluated following an intra-patient cross-validation method, achieving specificity and sensitivity results higher than 98%. Figure 11 and Figure 12 show the synthetic RGB representations of the HS cube and the corresponding thematic maps obtained when using this algorithm for normal brain and brain affected by cancer, respectively. In the thematic maps, the tumor tissue is represented in red color, the normal tissue in green, the hypervascularized tissue in blue and the background in black. Furthermore, in [171], this algorithm was fine-tuned, implemented onto the fully working demonstrator and qualitatively validated by using a test set of seven intraoperative HS images from four different patients. The classification results obtained demonstrated the capabilities of HSI in the identification of different types of tumors, not only high-grade gliomas. In addition, in this study it was demonstrated that no false positives were found when classifying HS images of normal brain not affected by cancer. Moreover, these HS images were processed in approximately 1 min, achieving surgical-time processing, using a manycore platform (computing system composed of hundreds of independent processor cores, designed for high-performance parallel processing) [177,178,179,180]. However, other recent experiments published by Torti et al. and Florimbi et al. demonstrated that the use of graphic processing units (GPUs) could accelerate the processing of the data to only a few seconds [181,182,183].

Finally, in 2019, one of the latest studies related to the use of HSI for brain cancer presented a comparison between the use of SVM-based algorithms and deep learning approaches [165,166]. These experiments were carried out using only the glioblastoma tumor samples available in the database (26 HS images from 16 patients) and taking into account the inter-patient variability following a leave-one-patient-out cross-validation. The results obtained using deep learning architectures were highly promising, improving the accuracy of the tumor identification by ~16% with respect to the SVM-based algorithm results. However, these studies require a higher amount of data in order to validate the results obtained so far and also a clinical validation of the system should be carried out to assess it. In addition, the use of the NIR information should be studied in order to evaluate whether it can be beneficial for the improvement of the classification results.

### 4.5. Medical HSI for Cancer Analysis Summary

In summary, Table 1 details the previously described studies performed on the use of HSI for in-vivo and ex-vivo cancer analysis. This table is organized as follows: (1) the type of cancer that has been investigated; (2) the type of sample involved in each study; (3) the HSI technology employed to obtain the images; (4) the data processing algorithms employed and the goals of the work; and (5) the subject of the study. 

## 5. Discussion

Several questions that remain unanswered as we look back at hyperspectral imaging for cancer detection and analysis can be asked again. The best HS sensor for the clinical adoption of HSI necessary to make the leap from basic research to clinical translational medicine is unknown and debatable. The sensor is likely to be task specific. We have reviewed that line-scanning pushbroom HS acquisition systems produce higher spectral resolutions, but spectral scanning HS cameras allow for higher spatial resolution. For example, in clinical cases where real-time is not a critical issue but a higher spectral resolution is required, a pushbroom HS camera is appropriate, but in cases where there is need for faster acquisition and where fewer spectral bands are required, spectral scanning or snapshot HS cameras would work well. Traditionally, regression-based algorithms may yield optimal performance and accuracy using only spectral signatures as inputs when the HS sensor exhibits high spectral resolution. However, there are certain tasks that also require interpretation of spatial information, and these types of tasks may require the use of a CNN or other method for contextual spatial information along with the spectral signatures.

Regardless of the sensors and HS acquisition system implemented, it is yet unknown which wavelength range of the electromagnetic spectrum is optimal for each application, and this could very well be again task specific. For example, push-broom technology with higher spectral resolution is suitable for the medical HSI research field, but it is important to acknowledge that medical HSI is still in the research phase. Most of the works reviewed in this article have been performed in the range of 400 to 1000 nm, broadly. Currently, as discussed in the HS sensor section, we appear to be lower-limited to 400 nm in the short wavelength range, but in the NIR and IR (infrared) range, it is possible to extend beyond 1000 nm, as some works have investigated. As reviewed in Section 3, the optical properties of biological tissue vary in spectra. It could be useful to have HS cameras below 400 nm for tasks sensitive to FAD (flavin adenine dinucleotide) or NADH. On the other hand, some tasks might require extending into the SWIR range for fat, water, or collagen analysis. Additionally, with regards to the broad-band spectrum, different algorithms that have been previously deployed and validated on other parts of the spectrum may not be generalizable in different ranges. It may happen that some algorithms work better compared with others for certain parts of the spectrum. For example, deep learning may have the potential to learn and tolerate more noise in the input signals, so a spectral range that contains more noise, either organic or systemic from the sensor, could be handled better by a CNN [140]. However, deep learning can be prone to over-fitting if there is insufficient data, and false negatives or positives can sometimes be predicted with exceptionally high confidence that the prediction is correct, which can lead to difficulties in interpreting the results. Therefore, it is conceivable that there are scenarios where traditional methods, such as SVM, may suppose the best choice for some specific works.

There has been a wide variety of works performed both ex-vivo and in-vivo, but there are challenges, both known and unknown, in moving from the former to the latter. Mainly, it is unknown whether ex-vivo HS data correlates well with in-vivo data. If an algorithm is trained on ex-vivo data, there is no guarantee that the algorithm can be generalizable enough to apply to in-vivo data for testing or, more doubtfully, for clinical use. What is more, it cannot be assured that the same algorithms would work for the same task when moving from ex-vivo to in-vivo. For example, the in-vivo data collection could degrade the quality of the HS data or induce artifacts from the patient and surgical environment, and this could result in a certain family of algorithms being less effective than others. Therefore, if this phenomenon was observed, then entire datasets of large numbers of ex-vivo patient data need to be re-collected if the system is adopted for in-vivo clinical use.

Currently, several systematic limitations of general HSI hinder its use in the operating theater or in the clinic. The most obvious one from a user perspective will be the size of the machinery and apparatus. The imaging time, which can be up to one minute, can be another limitation. With the increased imaging time, there is more room for artifact induction due to reasons such as patient movement or instrumentation movement. Furthermore, HSI can only be used to capture images of surfaces since this technology is not able to penetrate more than 1 mm depth in the tissue. On the other hand, the intra- and inter-patient variability in the spectral signatures of the same tissue can be a limitation for the classification methods, requiring a large quantity of data to generalize the processing methods. In addition, there are several potential problems when imaging biological tissue. Because the light sources are so close to the HS sensors and the biological tissue is often wet, there is a problem with specular glare. The human body is not topographically uniform, so imaging a small area with changes in elevation can create inconsistent illumination of the scene or cast shadows. Aside from inconsistencies with illumination, this could lead to issues with the HS camera focusing on multiple imaging planes. It is difficult to forecast the effects of this on large-scale HSI, and this could lead to issues with reproducibility or false negative/positive results because of insufficient HS quality to produce an accurate and reliable result. A level of quality control is necessary where the algorithms can automatically detect if the scene is not correctly captured to make an accurate prediction.

## 6. Conclusions

In summary, the reviewed studies present promising results for a wide variety of cancer detection applications based on medical imaging and for surgical guidance. However, currently we are limited in the field of HSI by technology restrictions and small datasets. The preliminary results warrant further research across all organ systems to determine if HSI has a place in the operating theater or in the clinic. Since HSI is non-contact, non-ionizing, non-invasive, and label-free, it is an attractive imaging modality with great potential. Most importantly, like all future translational technologies, HSI needs to be evaluated to demonstrate that it can be reliable, reproducible, and generalizable before it takes its place in medicine.

## Figures and Tables

**Table 1:** Summary of the state-of-the-art studies on the use of HSI for cancer analysis.

Reference	Year	Type of Cancer	Type of Sample	Spectral Range (nm)	Image Size (pixels)	# Bands	Light Source	Acquisition Mode	Algorithms ^¥^	Goal	Subject *
[123]	2007	Breast	in-vivo	450–700	1024 × 1528	34	InGaN LEDs	LCTF	Custom Algorithm	Classification	A
[142]	2011	Oral	in-vivo	450–650	350 × 350	48	Halogen	Snapshot	-	-	H
[143]	2011	Oral	in-vivo	400–700	-	40	Halogen	Snapshot	PCA, LDA	Dimensional reduction, Classification	H
[116]	2011	Gastric	ex-vivo	1000–2500	-	239	Halogen	Pushbroom	SVM, Integral Method, NDCI	Classification, Margin delineation	H
[184]	2012	Prostate	in-vivo	450–950	1392 × 1040	251	Xenon	LCTF	LS-SVM	Classification	A
[146]	2012	Tongue	in-vivo	600–1000	1392 × 1040	81	Halogen	AOTF	SR, SVM, RVM	Classification	H
[185]	2012	Prostate	in-vivo	500–950	1392 × 1040	251	Xenon	LCTF	LS-SVM	Classification	A
[117]	2013	Gastric	ex-vivo	400–800	640 × 480	72	Halogen	-	Cutoff point	Optimal wavelength selection, Classification	H
[125]	2013	Breast	ex-vivo	380–720	-	101	Xenon	-	Polynomial SVM	Automatic ROI detection based on contrast and texture information	H
[126]	2013	Breast	ex-vivo	380–720	-	101	Xenon	-	Fourier coefficient selection features, mRMR, RBF SVM	Feature extraction, Dimensional reduction, Classification	H
[124]	2014	Breast	in-vivo	500–600	1392 × 1040	26	Halogen	LCTF	Gabor Filter, Expectation Maximization	Microvessel sO_2_ segmentation & classification	A
[132] [133]	2014	H&N	in-vivo	450–950	1392 × 1040	251	Xenon	LCTF	Tensor Decomposition, PCA, KNN	Feature extraction, Classification	A
[134]	2014	H&N	in-vivo	450–950	1392 × 1040	251	Xenon	LCTF	PCA, FFD	Surgical margin delineation and in-vivo/in-vitro registration	A
[136]	2015	H&N	in-vivo	450–950	1392 × 1040	226	Xenon	LCTF	mRMR, KNN	Glare removal, Feature extraction, Automatic classification	A
[135]	2015	H&N	in-vivo	450–950	1392 × 1040	226	Xenon	LCTF	mRMR, RBF SVM, Chan-Vase active contour method	Glare removal, Feature extraction, Automatic classification, Active contour refinement	A
[118]	2015	Gastric	ex-vivo	400–800	480 × 640	81	Halogen	-	Mahalanobis distance, Cutoff point	Optimal wavelength selection, Classification	H
[145]	2016	Oral	in-vivo	390–680	-	30	-	-	RF	Classification	H
[78]	2016	Oral	in-vivo	390–680	1388 × 1040	30	Xenon	-	Customized	Image filtering (honeycomb pattern removal)	H
[120]	2016	Colon	in-vivo	405–665	585 × 752	27	Xenon	Filter Wheel	Recursive divergence, SVM	Wavelength selection, Classification	H
[137]	2016	H&N	in-vivo	450–950	1392 × 1040	251	Xenon	LCTF	SVM, MSF	Classification & segmentation	A
[144]	2016	Oral	in-vivo	390–680	1388 × 1040	30	Xenon	-	NCC, MNF, RF	Image registration and denoising, Glare detection, Classification	H
[140]	2017	H&N	ex-vivo	450–950	1392 × 1040	91	Xenon	LCTF	CNN, SVM, KNN, LR, DTC, LDA	Classification	H
[138]	2017	H&N	ex-vivo	450–50	1392 × 1040	91	Xenon	LCTF	Ensemble LDA	Classification	H
[139]	2017	H&N	ex-vivo	450–950	1392 × 1040	91	Xenon	LCTF	LDA, QDA, Ensemble LDA, Linear SVM, RBF SVM, RF	Classification	H
[119]	2019	Colon	ex-vivo	400–1000 900–1700	1 × 1312 1 × 320	-	Halogen	Pushbroom	Quadratic SVM	Classification	H
[186]	2019	H&N	ex-vivo	450–950	1392 × 1040	91	Xenon	LCTF	Inception CNN	Binary and Multiclass Classification	H
[173]	2016	Brain	in-vivo	400–1000 900–1700	1 × 1004 1 × 320	826 172	Halogen	Pushbroom	SVM, RF, ANN	Classification	H
[169]	2016	Brain	in-vivo	400–1000	1 × 1004	826	Halogen	Pushbroom	RF	Pre-Processing and Classification	H
[170]	2017	Brain	in-vivo	400–1000 900–1700	1 × 1004 1 × 320	826 172	Halogen	Pushbroom	tSNE, FR-tSNE STF, DCT-STF	Dimensional Reduction and Classification	H
[164] [171]	2018	Brain	in-vivo	400–1000	1 × 1004	826	Halogen	Pushbroom	SVM, FR-tSNE/PCA, KNN Filter, K-Means, MV	Classification	H
[165] [166]	2019	Brain	in-vivo	400–1000	1 × 1004	826	Halogen	Pushbroom	CNN, DNN, SVM, KNN Filter, K-Means	Binary and Multiclass Classification	H

* Subject: (H) Human; (A) Animal. ^¥^ Algorithms: (PCA) Principal Component Analysis; (LDA) Linear Discriminant Analysis; (SVM) Support Vector Machine; Normalized Cancer Index (NDCI); (LS-SVM) Least-Squares Support Vector Machine; (SR) Sparse Representation; (RVM) Relevance Vector Machine; (mRMR) maximal Relevance and Minimal Redundancy; (RBF) Radial Basis Function; (RF) Random Forest; (MSF) Minimum-Spanning Forest; (NCC) Normalized Cross-Correlation; (MNF) Minimum Noise Fraction; (CNN) Convolutional Neural Network; (KNN) K-Nearest Neighbor; (LR) Linear Regression; (DTC) Decision Tree Classification; (QDA) Quadratic Discriminant Analysis; (ANN) Artificial Neural Network; (tSNE) t-Distributed Stochastic Neighbor Embedding; (FR-tSNE) Fixed Reference t-Distributed Stochastic Neighbor Embedding; (STF) Semantic Texton Forests; (DCT-STF) Discrete Cosine Transform based Semantic Texton Forest; (MV) Majority Voting; (DNN) Deep Neural Network.
